# Anatomical conditions and patient-specific locked navigation templates for transverse sacroiliac screw placement: a retrospective study

**DOI:** 10.1186/s13018-020-01752-0

**Published:** 2020-07-13

**Authors:** Chao Wu, Jiayan Deng, Jian Pan, Tao Li, Lun Tan, Dechao Yuan

**Affiliations:** 1 Orthopedics Center of Zigong Fourth People’s Hospital, No. 19, Tanmulin street, Ziliujing district, Zigong, Sichuan Province China; 2 Digital Medical Center of Zigong Fourth People’s Hospital, Zigong, China

**Keywords:** Sacral anatomy, Sacral fracture, TSS, Minimally invasive, 3D printing

## Abstract

**Objective:**

To analyse the anatomical conditions of transverse sacroiliac screw (TSS) about the S1 and S2 segments in order to develop and validate a locked navigational template for TSS placement.

**Methods:**

A total of 22 patients with sacral fractures were involved in this study from May 2018 to February 2019. Patients were divided into two groups according to the surgery procedure: locked template group and conventional group. The CT data of 90 normal sacra were analysed. The long axis, short axis and lengths of TSS, cancellous corridors were measured through 3D modelling. A patient-specific locked navigation template based on simulated screws was designed and 3D printed and then used to assist in TSS placement. The operative time and radiation times were recorded. The Matta criteria and grading score were evaluated. The entry point deviation of the actual screw placement relative to the simulated screw placement was measured, and whether the whole screw was in the cancellous corridor was ob`served.

**Results:**

S1 screws with a diameter of 7.3 mm could be inserted into 69 pelvises, and S2 screws could be inserted in all pelvises. The S1 cancellous corridor had a long axis of 25.44 ± 3.32 mm in males and 22.91 ± 2.46 mm in females, a short axis of 14.21 ± 2.19 mm in males and 12.15 ± 3.22 mm in females, a corridor length of 153.07 ± 11.99 mm in males and 151.11 ± 8.73 mm in females, and a proportional position of the optimal entry point in the long axis of the cancellous corridor of 35.96 ± 10.31% in males and 33.28 ± 7.2% in females. There were significant differences in the corridor long axis and corridor short axis between sexes (*p* < 0.05), and there were no significant differences in corridor length and proportional position of the optimal entry point in the long axis of the cancellous corridor between sexes (*p* > 0.05). The S2 cancellous corridor had a long axis of 17.58 ± 2.36 mm in males and 16 ± 2.64 mm in females, a short axis of 14.21 ± 2.19 mm in males and 13.14 ± 2.2 mm in females, a corridor length of 129.95 ± 0.89 mm in males and 136.5 ± 7.96 mm in females, and a proportional position of the optimal entry point in the long axis of the cancellous corridor of 46.77 ± 9.02% in males and 42.25 ± 11.95% in females. There were significant differences in the long axis, short axis and corridor length (*p* < 0.05). There was no significant difference in the proportional position of the optimal entry point in the long axis of the cancellous corridor (*p* > 0.05). A total of 20 transversal sacroiliac screws were successfully implanted into 10 patients with the assistance of locked navigation templates, and a total of 24 transversal sacroiliac screws were successfully implanted into 12 patients under C-arm fluoroscopy. There was a significant difference in surgical time (88 ± 14.76 min vs 102.5 ± 17.12 min, *p* = 0.048), radiation times (11.5 ± 1.78 vs 54.83 ± 6.59, *p* < 0.05) and screw grading between two groups (nineteen screws in grade 0, one screw in grade 1 and 0 screws in grade 2 vs fourteen screws in grade 0, 8 screws in grade 1 and 2 screws in grade 2, *p* = 0.005). All screw entry point deviations were shorter than the short axis of the cancellous corridor, and all screws were located completely within the cancellous corridor.

**Conclusion:**

Approximately 76% of males and females can accommodate screws with diameters of 7.3 mm in S1, and all persons can accommodate the same screw in S2. From the standard lateral perspective of the sacrum, the optimal entry point of the transverse screw is in the first 1/3 of the cancellous corridor for S1 and the centre of the cancellous corridor for S2. The patient-specific locked navigation template assisted in TSS placement with less operative time, less intraoperative fluoroscopy and higher safety of screw placement compared with traditional surgery.

## Introduction

Since the posterior pelvic ring disorders have a high risk of sacral nerve root injuries and high-grade biomechanical instability, the traditional surgical treatment method is an internal fixation by plate, including posterior percutaneous plate fixation and anterior open reduction plate screw fixation. However, these methods are associated with poor pelvic stability, plate or screw loosening, fracture reduction failure, large trauma, wound infection and other risks [1, 2]. Matta and Saucedo [3] discovered that internal sacroiliac screw fixation for unstable pelvic ring fractures can significantly reduce trauma; it has subsequently become a popular technique for a large number of orthopaedic surgeons. Compared with traditional sacroiliac screws, transverse sacroiliac screws (TSS) have better pelvic stability due to the longer screw corridor [4], but TSS are not for everyone. According to the anatomical analysis of 76 normal pelvic CT cases by König [5] et al., it was found that in 37% of males, the S1 osseous corridor was unable to accommodate a screw with a diameter of 8 mm, compared with 34% of females. All male S2 corridors can accommodate screws with a diameter of 8 mm, while 13% of female S2 corridors cannot. A large number of studies have shown that the anatomical morphology of the sacrum has a significant effect on the placement of transverse screws [6–9], but the anatomical conditions for TSS in patients of Asian ethnicity have been less reported.

In the internal fixation of TSS, the insertion of a hollow screw guide wire is very important, and placement under traditional fluoroscopy not only increases the radiation dose for the doctor and patient but also requires the doctor’s detailed knowledge, clinical experience, anatomical familiarity and X-ray interpretation ability [10, 11]. Screw penetration rates have been reported to be as high as 68%, and nerve injury has been reported in as many as 7.9% of screw placements by fluoroscopy [12–14]. With the application of various navigation equipment, the safety and accuracy of screw placement have been greatly improved. However, there is a learning process required before they can be used proficiently, which adds some difficulties for the doctors [15–17]. In addition, the acquisition of navigation equipment is expensive and becomes an economic burden for most hospitals.

To solve the above problems, this study analysed and measured the cancellous corridor of TSS through digital 3D modelling and provided the anatomical conditions for the placement of TSS in a population in Southwest China. For patients with posterior pelvic ring disorders in our hospital, a personalized navigation template was then designed, 3D printed and applied for intraoperative application. After the surgery, the screws were evaluated for penetration into the cortex. The deviation of the screws from the optimal insertion point at the entrance to the sacrum was measured, thereby verifying the safety and accuracy of sacroiliac screw placement assisted by personalized navigation template.

The purpose of this study is as follows: (i) to discuss the feasibility of transverse screws about the S1 and S2 segments; (ii) to measure the optimal screw entry point and safety screw corridor of transverse screws about the S1 and S2 segments through a digital 3D model; (iii) to design a locked, patient-specific navigation template for TSS placement; and (iv) to evaluate the accuracy and safety of the locked navigation template for TSS placement.

## Materials and methods

### Measures of anatomical conditions

A total of 47 adult males and 43 adult females who underwent computed tomography (CT) scans of their pelvises between January 2017 and May 2018 were included in the study. Investigations that revealed obvious traumatic, degenerate or osteolytic conditions were excluded. Investigations with a slice thickness of 0.625 mm were selected. Institutional review board approval was obtained.

**Fig. 1:**
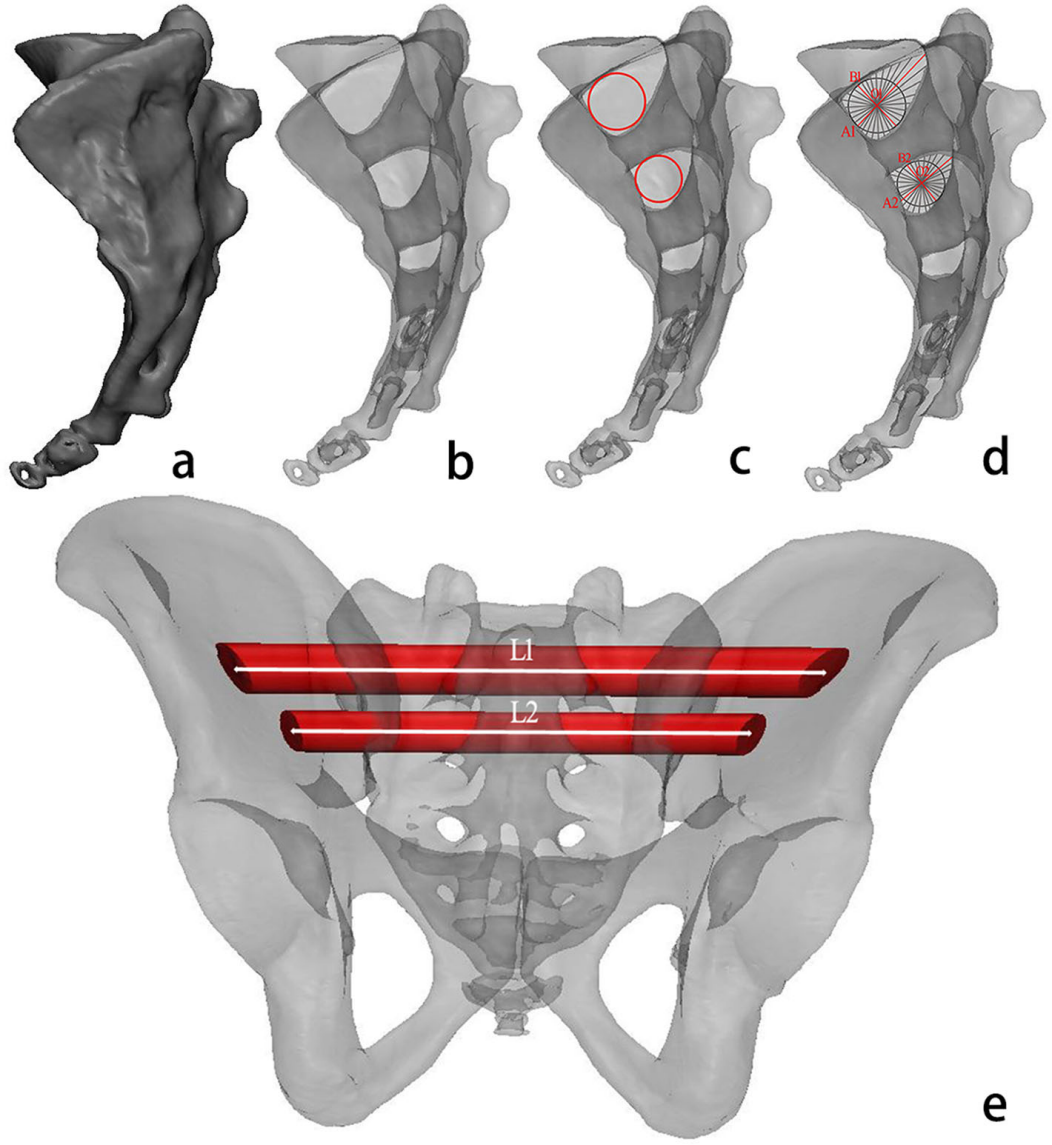
Measurements of anatomical conditions: **a**. 3D model of the sacrum; **b**. 3D model of the sacrum in transparent view; **c**. Maximum inscribed circle in the secure corridor; **d**. Lines through the centre of the maximum inner circle in the cancellous corridor (grey line), the long axis of the corridor (A, red line) and the short axis of the corridor (B, red line); **e**. Total corridor length, marked with a white line

The CT data were imported into Mimics 21.0 (Materialise, Leuven, Belgium). The CT threshold of bone was measured, the sacral cortex was extracted and a 3D model of the sacrum was constructed (Fig. 1a). In the transparent view (Fig. 1b), the maximum inscribed circle of the cancellous corridor was marked (Fig. 1c). Drawing lines through the centre of the circle, the longest line was considered the long axis of the corridor (A), and the shortest line was regarded as the short axis of the corridor (B) of the cancellous corridor (Fig. 1d). The centre of the maximum inner circle (O) is considered the optimal screw entry point, and the proportional position of the optimal entry point on the long axis of the corridor was calculated (Fig. 1d). The total corridor length (L1 and L2) was the distance from the centre of the maximum inscribed circle to the projection point on the left pelvic surface and to the projection point on the right (Fig. 1e).

### Patients

We retrospectively identified patients treated with TSS from May 2018 to February 2019 and enrolled in our trauma centre’s database. The inclusion criteria were as follows: definite sacral fracture with Denis type I/II, reducible sacroiliac dislocation, Tile classification B/C, and age 18 to 60 years old. The exclusion criteria were as follows: patients with severe systemic diseases or open pelvic injuries.

A total of 22 patients with sacral fractures were involved in this study from May 2018 to February 2019. Patients were divided into two groups according to the surgery procedure: locked template group and conventional group. Ten patients were involved in the locked template group and 12 patients were enrolled in the conventional group (Table 1). The operations were performed by the same surgeon with more than 10 years of experience. All patients were informed of the experimental design before the surgery and signed an informed consent form.

**Table 1:** General information of patients

Variables	Locked template group	Conventional group	Statistics	*p* value
Number of patients	10	12	—	—
Age (mean ± SD)	42.1 ± 12.7	41.07 ± 3.99	0.318	0.754
Gender (*N*)				
Male	4	5	0.006	0.937
Female	6	7		
BMI (body mass index, kg/cm^2^)	23.73 ± 1.98	25.03 ± 2.10	− 1.487	0.153
Trauma causes (*N*)				
Motor vehicle accident	7	8	0.086	0.958
High-energy fall	2	3		
Other injury	1	1		
Type of posterior pelvic ring disruption (*N*)				
Denis I fracture	5	6	0.052	0.974
Denis II fracture	3	4		
SI dislocation	2	2		
Tile classification (*N*)				
B2	8	9	0.078	0.781
C1	2	3		

*N* number, “—” not included

### Design of the locked navigation template

**Fig. 2:**
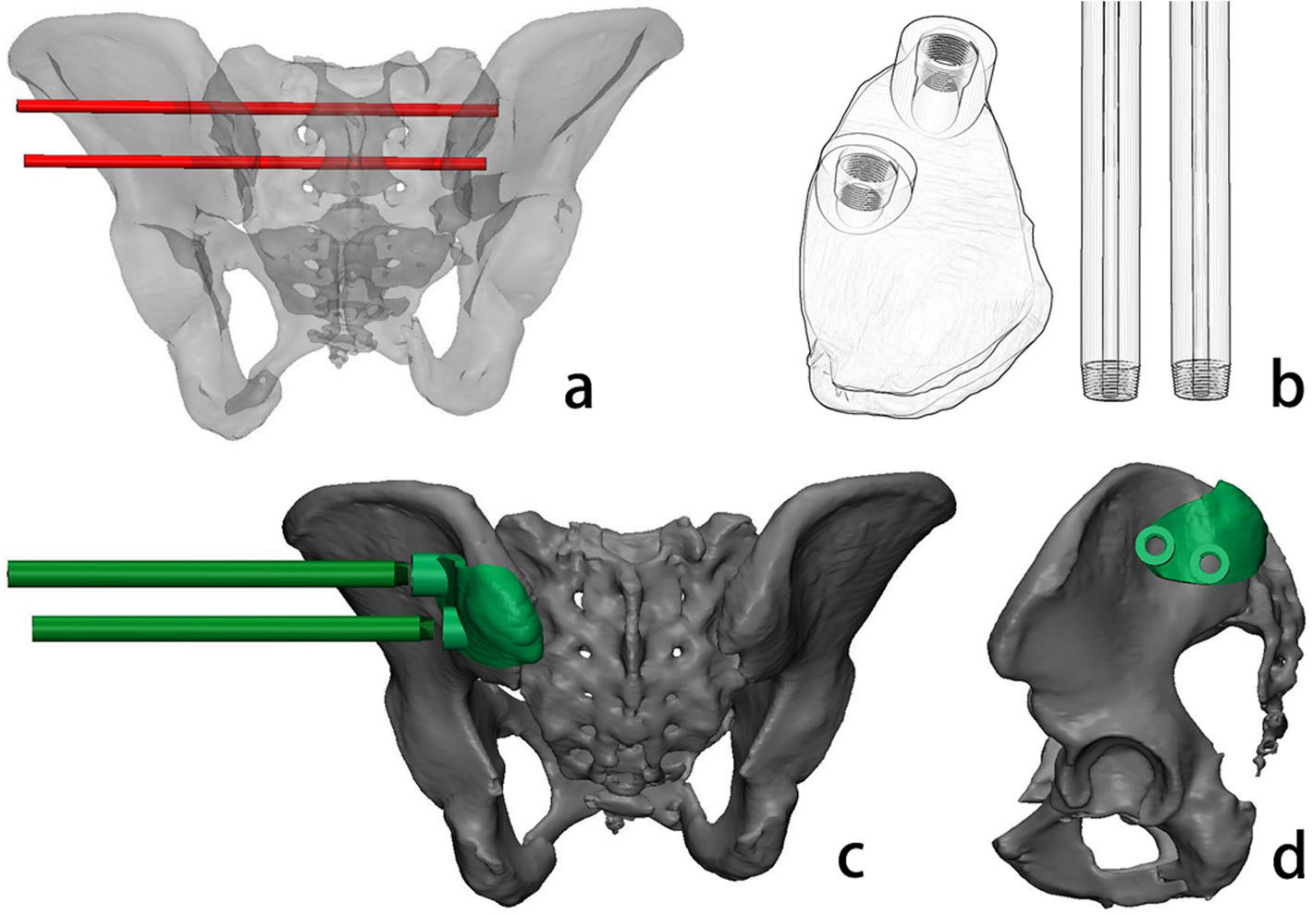
Simulated screw placement and design of the locked navigation template: **a**. simulated placement of the transverse sacroiliac screw in back view; **b**. sketch of the locked navigation template; **c**. locked navigation template base and guide tube from the back pelvic view; **d**. locked navigation template base from the lateral pelvic view

The CT images of the patients were imported into Mimics 21.0, and a 3D model of the sacrum and pelvis was built. In accordance with the above method, the virtual screw was inserted from the optimal screw entry point and then passed along the optimal screw corridor to complete the simulated screw placement (Fig. 2a). The pelvis and virtual screws were imported into 3-matic 13.0 (Materialise, Leuven, Belgium). The guide pipe and locked template were designed according to the direction of the virtual screw (Fig. 2b), and the inside of the guide pipe was designed with a diameter of 2.6 mm for passing the 2.5 mm K-wires. The base of the locked navigation template was designed according to the shape of the pelvic surface where the virtual screw is on (Fig. 2c) and designed with a 9 mm diameter pipe for passing screws (Fig. 2d).

### Preoperative preparation

**Fig. 3:**
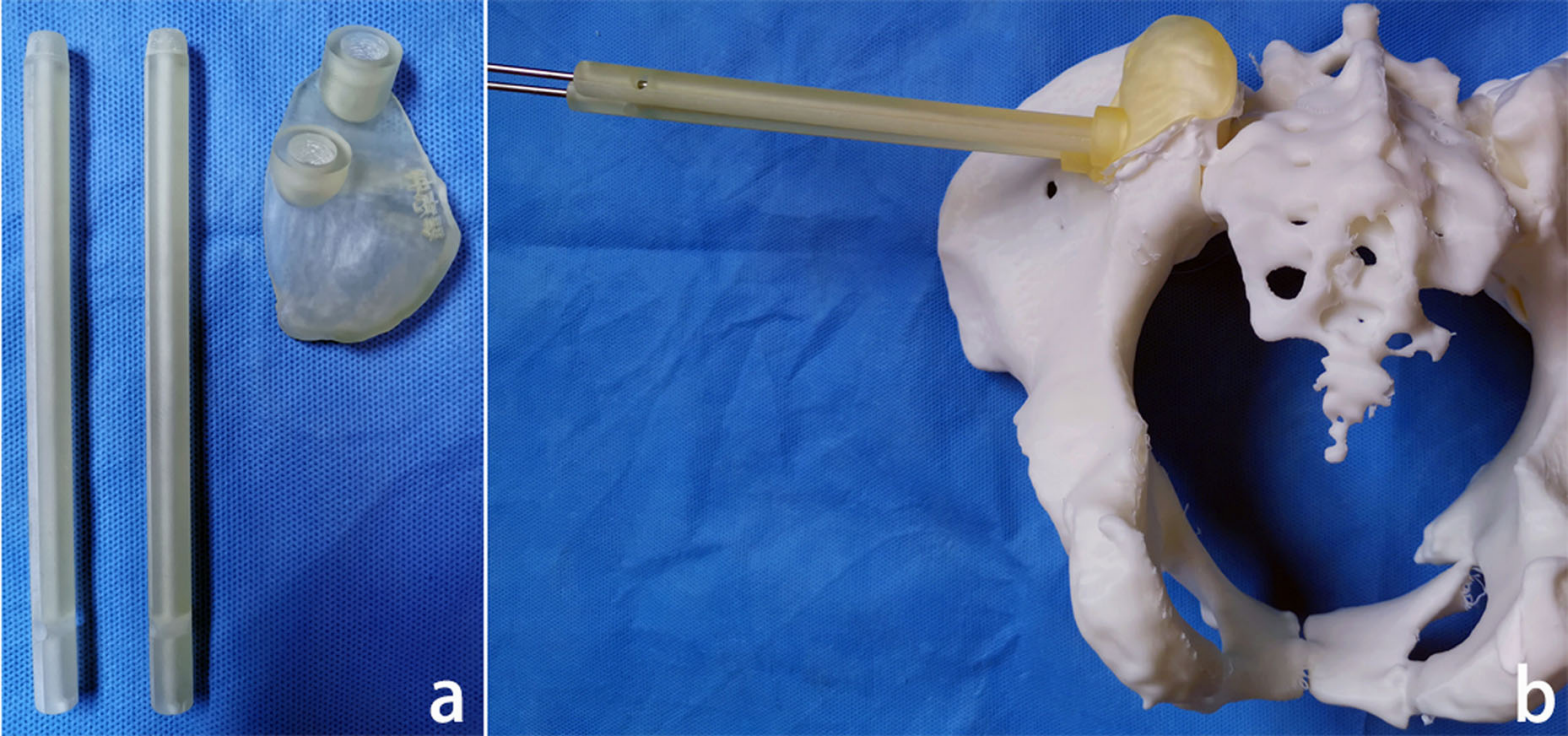
Preoperative preparation of the locked navigation template: **a**. 3D-printed locked navigation template; **b**. guide pipe screwed into the base and K-wires implanted through the guide pipe

The pelvic model and navigation template were printed (Fig. 3a). Then, the base of the template was attached to the posterior superior iliac spine, the guide pipe was screwed into the base, and K-wires with a diameter of 2.5 mm were implanted along the guide pipe (Fig. 3b). Whether the K-wires broke through the bone cortex was observed, and the consistency of the K-wires’ placements with the preoperative design was evaluated. The locked navigation template was sterilized at low temperatures before operation.

**Fig. 4:**
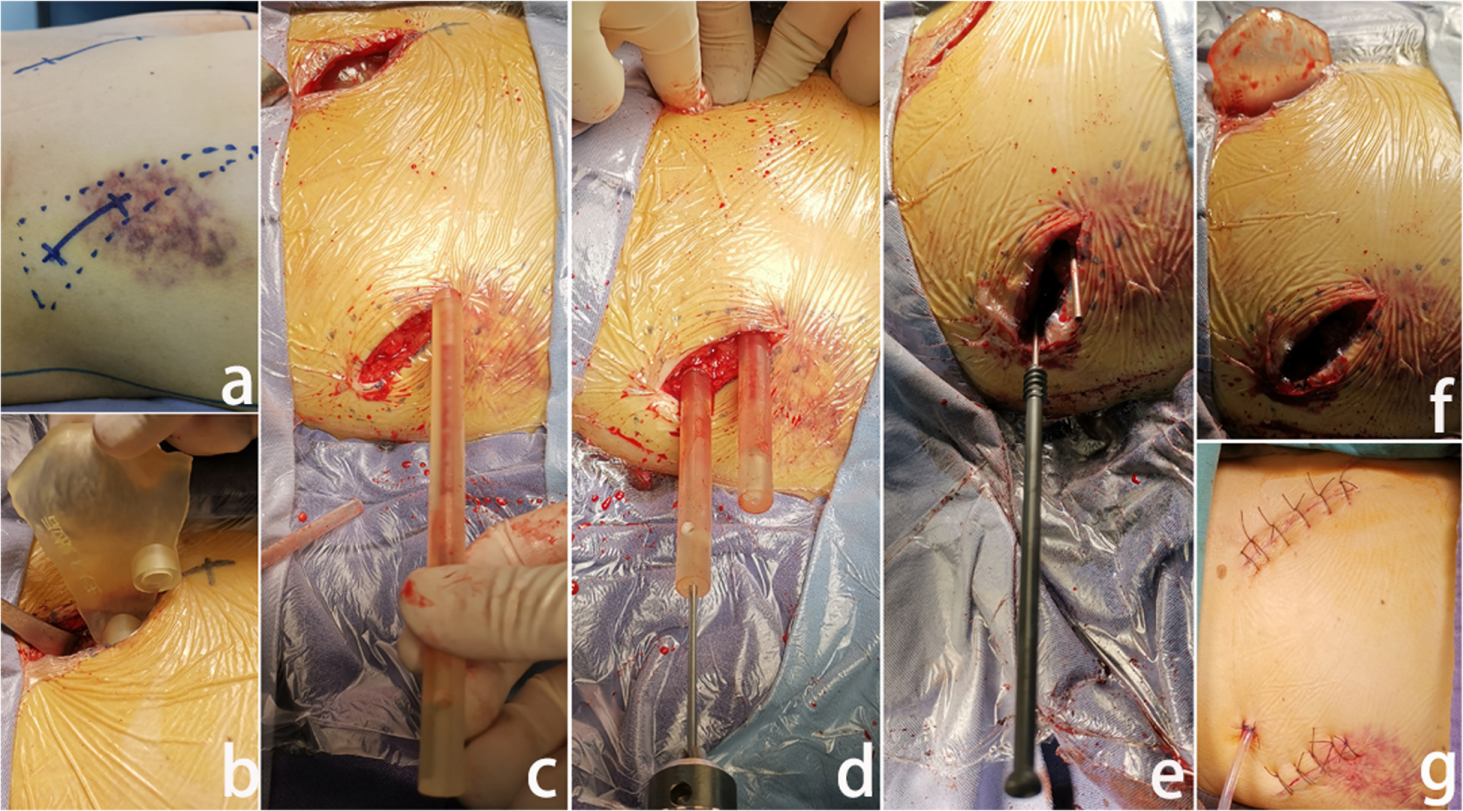
A case of sacral fracture in a 42-year-old female patient: **a**. Preoperative screw holes were localized on the body surface; **b**. The template base was inserted; **c**. A small incision was made, and threaded guide pipes were inserted; **d**. The guide pipes were screwed onto the base, and 2 K-wires were implanted through the guide pipes; **e**. The guide pipes were removed, and a hollow screw was placed; **f**. The K-wires and template base were removed; **g**. The condition of the postoperative wound is displayed

### Surgical technique

Under general anaesthesia, the patient was placed in the prone position as recommended for the surgical procedure on a radiolucent operation table. In most cases, internal fixation of the anterior pelvic ring was performed with indirect reduction.

#### Locked template group

First, the posterior superior iliac spine and the TSS placement points were located on the body surface (Fig. 4a). The incision of the posterior superior iliac spine was performed according to the size of the navigation template base, and soft tissue was stripped from the bones. The template base was inserted through the incision and completely attached to the corresponding anatomical positions on the posterior superior iliac spine (Fig. 4b). Second, a small incision was made at the location of the TSS entry points, through which the threaded guide pipes were inserted and screwed onto the base (Fig. 4c). Third, 2 K-wires with a 2.5 cm diameter were implanted into the sacrum through the guide pipes. Fluoroscopy of the pelvic inlet and outlet and the lateral sacrum were performed to confirm the position of the K-wires (Fig. 4d). Then, the guide pipes were removed, and a hollow screw with a 7.3 mm diameter was screwed into the sacrum along the K-wires through the iliac bone (Fig. 4e). Fifth, fluoroscopy was performed again to confirm the screw positions. Finally, the K-wires and template base were removed (Fig. 4f, g).

#### Conventional group

The surgical procedure was roughly the same, except that the K-wires of the conventional group were inserted under C-arm fluoroscopy guidance, based on lateral, inlet and outlet views.

### Evaluation criteria

#### Normal anatomical parameters of the sacrum

The 90 pelvises were divided into two groups by gender. The long axis, short axis and length of the cancellous corridor were measured, and the proportional position of the optimal entry point on the cancellous corridor was calculated.

#### Operated information for patients

The operation time and radiation times were recorded to evaluate the operation quality. The following grading score was used to evaluate the safety of the TSS [18]: grade 0 = safe placement, screws located in cancellous bone; grade 1 = cortical bone perforation less than 2 mm; and grade 2 = cortical bone perforation greater than 2 mm. Grade 0 and 1 placement are considered successful and safe, and grade 2 indicates the possibility of nerve damage.

The quality of the reduction was assessed by the Matta score [3]: excellent, less than 4 mm of fracture displacement; good, 4–10 mm of fracture displacement; and fair, 10–20 mm of fracture displacement.

#### Deviation of screw entry point

**Fig. 5:**
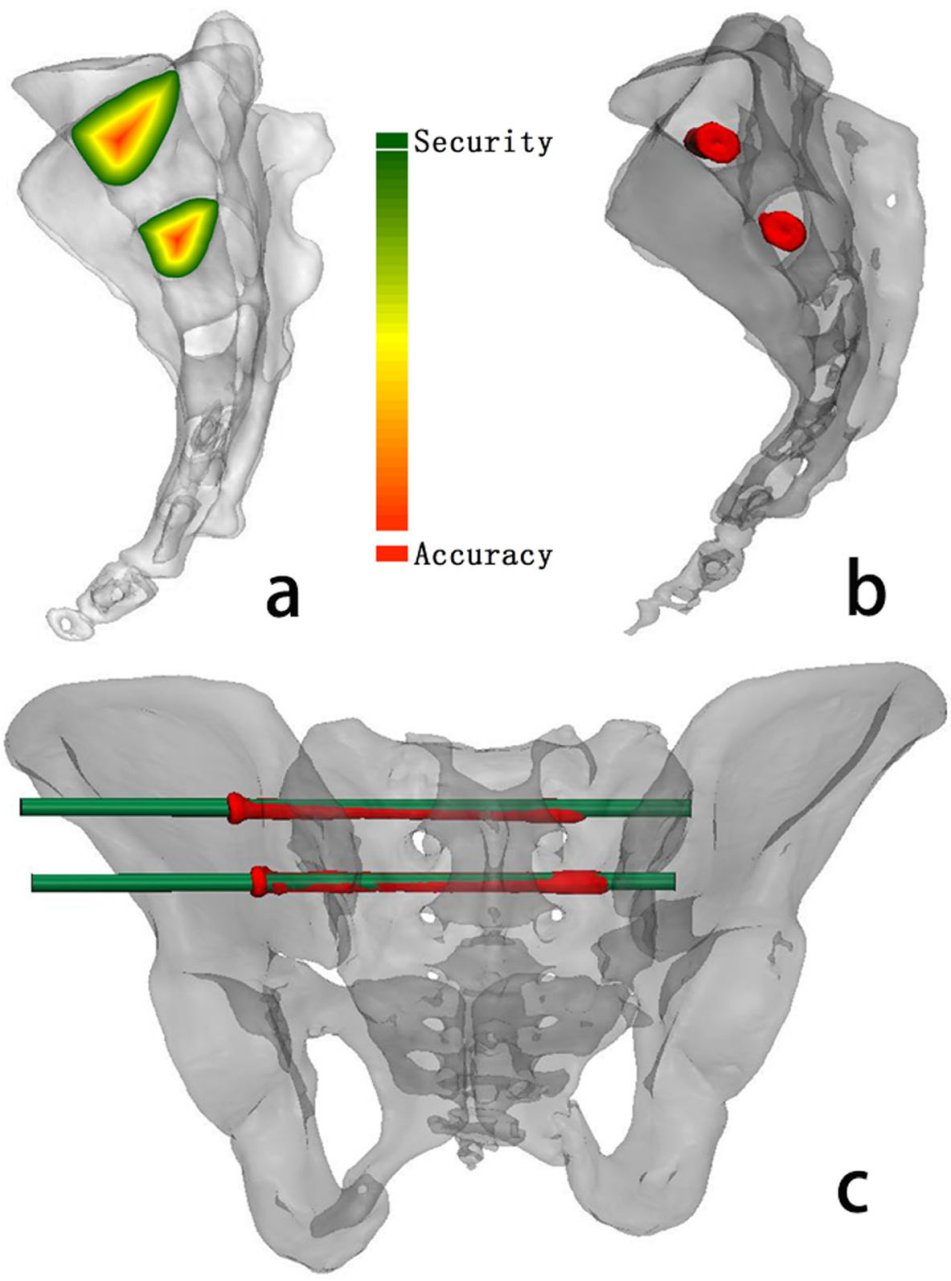
**a**. Distribution of the security index in S1 and S2 from the sagittal view. Red represents the area where the screw is placed accurately, which is close to the optimal screw corridor. Green represents the area where the screw can be placed safely but not in the optimal corridor. **b**. The whole screw is located in the safe corridor in a postoperative sagittal sacral view. **c**. The registered 3D model of the pelvis is shown from a posterior pelvic view

By registering the pre- and postoperative CT images, the distance between the actual and planned screws at the point of entrance was measured based on the 3D model [19] (Fig. 5b). On the sagittal view of the sacrum (Fig. 5c), the screw was evaluated if it was completely located within the cancellous corridor.

### Statistical analysis

All statistical analyses were performed in SPSS 19.0 (SPSS Inc.; Chicago, IL, USA). Independent sample T tests were performed for the long axis, short axis and length of the cancellous corridor and for proportional position of the optimal entry point in the cancellous corridor. Descriptive statistics were obtained for general and operative patient parameters.

## Results

### Measurements

The CT data of 90 normal adults were measured. S1 screws with a diameter of 7.3 mm could not be inserted in 21 patients; of those that could accept the screw, 36 were male and 33 were female. S2 screws with a diameter of 7.3 mm could be inserted into all pelvises, (47 males and 43 females). The distribution of the security index was obtained according to the shape of the cancellous corridor in S1 and S2 (Fig. 5a). The cancellous corridor for S1 had a long axis of 25.44 ± 3.32 mm in males and 22.91 ± 2.46 mm in females, which was significantly different between the sexes (*p* = 0.001). For S2, the long axis was 17.58 ± 2.36 mm in males and 16 ± 2.64 mm in females, which was significantly different between the sexes (*p* = 0.004). The cancellous corridor for S1 had a short axis of 14.01 ± 3.53 mm in males and 12.15 ± 3.22 mm in females, with a significant difference between the sexes (*p* = 0.025); for S2, the short axis was 14.21 ± 2.19 mm in males and 13.14 ± 2.2 mm in females, with a significant difference between the sexes (*p* = 0.023). The cancellous corridor for S1 had a length of 153.07 ± 11.99 mm in males and 151.11 ± 8.73 mm in females, with no significant difference between the sexes (*p* > 0.05); for S2, it had a length of 129.95 ± 0.89 mm in males and 136.5 ± 7.96 mm in females, with a significant difference between the sexes (*p* < 0.05). The proportional position of the optimal entry point in the cancellous corridor was 35.96 ± 10.31% in males and 33.28 ± 7.2% in females for S1, with no significant difference between the sexes (*p* > 0.05), and 46.77 ± 9.02% in males and 33.25 ± 11.95% in females for S2, with no significant difference between the sexes (*p* > 0.05) (Table 2).

**Table 2:** Measurement of transverse sacroiliac screw cancellous corridor

S1	Parameter measured	Male (*n* = 36)	Female (*n* = 33)	*p* value
	Long axis of corridor (mm) (A)	25.44 ± 3.32	22.91 ± 2.46	0.001
	Short axis of corridor (mm) (B)	14.01 ± 3.53	12.15 ± 3.22	0.025
	Corridor length (mm) (L1)	153.07 ± 11.99	151.11 ± 8.73	0.438
	Proportional position of the optimum entry point to long axis of secure corridor width (%)	35.96 ± 10.31	33.28 ± 7.2	0.172
S2	Parameter measured	Male (*n* = 47)	Female (*n* = 43)	*p* value
	Long axis of corridor (mm) (A)	17.58 ± 2.36	16 ± 2.64	0.004
	Short axis of corridor (mm) (B)	14.21 ± 2.19	13.14 ± 2.2	0.023
	Corridor length (mm) (L2)	129.95 ± 0.89	136.5 ± 7.96	0.000
	Proportional position of the optimum entry point to long axis of secure corridor width (%)	46.77 ± 9.02	42.25 ± 11.95	0.065

### Clinical outcome

**Fig. 6:**
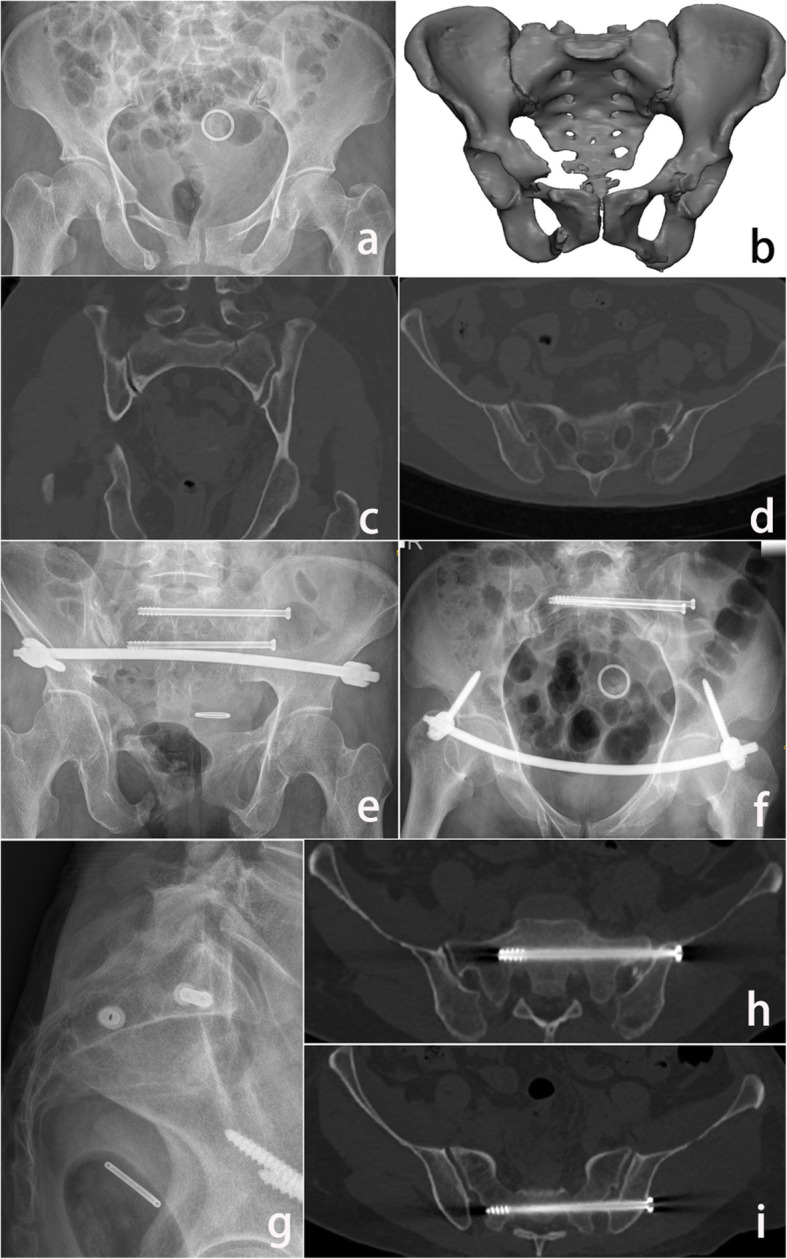
Preoperative and postoperative images of a 42-year-old female patient with sacral fracture: **a**. Preoperative anteroposterior radiograph images; **b**. Preoperative 3D images; **c**, **d**. Preoperative CT (coronary position and axial position) image showing sacral fracture; **e**, **f**. Postoperative pelvis radiograph (outlet/inlet view); **g**. Postoperative lateral position of the sacrum; **h**, **i**. Postoperative CT axial image confirming the placement of transverse sacroiliac screws about S1 and S2

Twenty TSS were successfully inserted into 10 patients with the assistance of locked navigation templates, with a surgical time of 88 ± 14.76 min and radiation times of 11.5 ± 1.78, and 24 TSS were inserted in 12 patients in the conventional group, with a surgical time of 102.5 ± 17.12 min and radiation times of 54.83 ± 6.59. There was a significant difference in surgical time (88 ± 14.76 min vs 102.5 ± 17.12 min, *p* = 0.048) and radiation times (11.5 ± 1.78 vs 54.83 ± 6.59, *p* < 0.05) between the two groups. There was a significant difference in screw grading between the two groups (nineteen screws in grade 0, one screw in grade 1 and 0 screws in grade 2 vs fourteen screws in grade 0, 8 screws in grade 1 and 2 screws in grade 2); in the locked template group, one screw broke through the cortex for the narrow screw corridor of S1; in the conventional group, it is difficult for the surgeon to adjust the position and orientation of the screw under fluoroscopy, which results in 10 screws breaking through the cortex. There was no significant difference in Matta scores between the two groups (Table 3). There were no postoperative complications of infection or nerve root injury in two groups. With a screw entry point average deviation of 1.95 ± 0.39 mm for S1 and 1.34 ± 0.65 mm for S2 in locked template group. All screw entry point deviations were shorter than the short axis of the cancellous corridor, and all screws were located within the cancellous corridor (Fig. 6).

**Table 3:** Comparison of the clinical outcome between two groups

Variables	Locked template group (*N* = 10)	Conventional group (*N* = 12)	Statistics	*p* value
Surgical time (min)	88 ± 14.76	102.5 ± 17.12	− 2.107	0.048*
Radiation exposure times (mean ± SD)	11.5 ± 1.78	54.83 ± 6.59	− 20.156	< 0.05*
**Matta criteria (*N*)**				
Excellent	8	9	− 0.272	0.785
Good	2	3		
Fair	0	0		
**Grading criteria (*N*)**				
Grade 0	19	14	− 2.779	0.005*
Grade 1	1	8		
Grade 2	0	2		
**Deviation of screw entry point (mm)**				
**S1**	1.95 ± 0.39	—	—	—
**S2**	1.34 ± 0.65	—	—	—

*Statistical significance

*N* number, “—” not included

## Discussion

### Anatomical conditions of the TSS

Transversal sacroiliac screw placement is often well planned because not all patients are able to accommodate one. Some investigators had previously evaluated and analysed the shape of the sacrum and the parameters of the cancellous corridor for the placement of TSS. Thomas et al. [8] proposed that the transverse corridor of S1 could accommodate an average of 3.7 screws of 7.3 mm in diameter. Radetzki et al. [20] found that 20% of pelvises could not allow the implantation of 7.3 mm transverse screws in S1, and 40% of pelvises could not allow the implantation of 7.3 mm transverse screws in S3. Mendel et al. [4] concluded that 18% of pelvises could not allow the implantation of 7.3 mm transverse screws in S1. However, these parameters are primarily anatomical and are rarely applied in surgery. In our study, we performed anatomic analysis of the pelvises of normal adults in southwestern China, converting the pelvis into a 3D visualization model to better demonstrate the shape and location of the TSS cancellous corridor. We measured the long axis and short axis of the cancellous corridor and found that 69 (77%) pelvises could accommodate screws with diameters of 7.3 mm in S1, and all pelvises could accommodate screws with diameters of 7.3 mm in S2. Furthermore, the diameter of the corridors in both S1 and S2 showed a significant difference between sexes. In addition, the location of the optimal entry point in the cancellous corridor was found. From the standard lateral perspective of the sacrum, the optimal entry point of the transverse screw was in the first 1/3 of the cancellous corridor for S1 and the centre of the cancellous corridor for S2.

### Advantages of 3D-printed guiding template

Although screw placement safety has greatly improved with the introduction of various navigation devices, some screws still break through the cortex. According to Zwingmann et al. [13], the nail-setting cortex has a breakthrough rate of 30.77% under computer navigation. According to Zwingmann et al. [21], under computer navigation, 19% of screws break through the cortex. The breakthrough rate of conventional nailing is as high as 50% [13, 21, 22]. Under 3D fluoroscopy navigation, the placement of screws has a 50% cortical breakthrough rate [23]. Liu et al. [24] used a 3D-printed navigation template to assist with the placement of sacroiliac screws, and the operation time and X-ray exposure were significantly reduced compared to percutaneous sacroiliac screws.

In recent years, there has been an increasing number of reports on 3D-printed navigation template-assisted screw placement, but there are few reports on 3D-printed locked navigation template-assisted TSS placement. Most of the 3D-printed navigation templates have been reported to be integrated structures, with no significant advantage in reducing intraoperative blood loss [24]. The 3D-printed navigation template in this study was designed in split form and locked as a whole through a threaded structure. During the operation, the base and guide pipe are placed separately, which can not only significantly reduce the size of the incision and blood loss but also effectively avoid the imprecision of screw placement caused by skin tension. In this study, the average deviation of the screw entry point was less than 2 mm and was within the mean of the short axis, and 100% of the screws were safely located within the cancellous corridor. One screw touched the cortical bone, but the patient showed no nerve injury symptoms. The average design time of a single locked navigation template is less than half an hour, and the average 3D printing time is 6 h. A single locked navigation template costs $140, which is significantly less than other navigation devices.

### Surgical tips

First, the soft tissue under the incision needs to be completely removed to ensure that the navigation template base can tightly attach to the bone. Second, the entry points of transversal sacroiliac screws should be marked on the body surface under fluoroscopy before the operation to reduce skin and muscle tension. Third, the base of the navigation template should be wrapped around the posterior superior iliac spine to prevent movement. Fourth, the guide pipes should be designed with a conical thread structure, which is conducive to assembly with the base during the operation. Last, together with the S1 and S2 screws, the stability of the pelvic ring can be maintained.

### Limitation

Some limitations of this study should be noted. First, although the screws were placed in the cancellous corridor in this study, there was still a deviation between the actual screw entry point and the simulated screw entry point. We will correct the navigation template in future studies to improve the accuracy of screw placement. Second, one patient’s S1 transverse screw destroyed the bone cortex, and the cause will be further tracked in a later study. Finally, only 10 patients were recruited in this study; the sample size will be expanded in a later study to further verify the safety and accuracy of 3D-printed navigation template-assisted placement of TSS.

## Conclusion

This study demonstrates that approximately two thirds of individuals can accommodate screws with diameters of 7.3 mm in S1 and that all individuals can accommodate the same screw in S2. Prior to placing TSS, accurate CT scan analysis of the sacrum must be performed in each patient. TSS placement with patient-specific locked navigation template assistance is clinically feasible and with less operative time, less intraoperative fluoroscopy and higher safety of screw placement compared with traditional surgery.

## Data Availability

The device(s) are FDA-approved or approved by the corresponding national agency for this indication. The patients’ data was authorized to our research.

## Abbreviations

TSS: Transverse sacroiliac screw
SI: Sacroiliac joint
